# Development and Validation of a Pathology Image Analysis-based Predictive Model for Lung Adenocarcinoma Prognosis - A Multi-cohort Study

**DOI:** 10.1038/s41598-019-42845-z

**Published:** 2019-05-03

**Authors:** Xin Luo, Shen Yin, Lin Yang, Junya Fujimoto, Yikun Yang, Cesar Moran, Neda Kalhor, Annikka Weissferdt, Yang Xie, Adi Gazdar, John Minna, Ignacio Ivan Wistuba, Yousheng Mao, Guanghua Xiao

**Affiliations:** 1 Quantitative Biomedical Research Center, Department of Clinical Sciences, University of Texas Southwestern Medical Center, Dallas, Texas 75390 USA; 2 Department of Bioinformatics, University of Texas Southwestern Medical Center, Dallas, Texas 75390 USA; 3 Department of Statistics, Southern Methodist University, Dallas, Texas USA; 4 Department of Pathology, National Cancer Center/Cancer Hospital, Chinese Academy of Medical Sciences and Peking Union Medical College, Beijing, 100021 China; 5 Department of Translational Molecular Pathology, The University of Texas MD Anderson Cancer Center, Houston, Texas USA; 6 Department of Thoracic Surgery, National Cancer Center/Cancer Hospital, Chinese Academy of Medical Sciences (CAMS), Beijing, China; 7 Department of Pathology, Division of Pathology/Lab Medicine, The University of Texas MD Anderson Cancer Center, Houston, Texas USA; 8 Simmons Comprehensive Cancer Center, University of Texas Southwestern Medical Center, Dallas, Texas USA; 9 Hamon Center for Therapeutic Oncology, University of Texas Southwestern Medical Center, Dallas, Texas USA; 10 Department of Pathology, University of Texas Southwestern Medical Center, Dallas, Texas USA; 11 Department of Internal Medicine, University of Texas Southwestern Medical Center, Dallas, Texas USA

**Keywords:** Cancer imaging, Statistical methods

## Abstract

Prediction of disease prognosis is essential for improving cancer patient care. Previously, we have demonstrated the feasibility of using quantitative morphological features of tumor pathology images to predict the prognosis of lung cancer patients in a single cohort. In this study, we developed and validated a pathology image-based predictive model for the prognosis of lung adenocarcinoma (ADC) patients across multiple independent cohorts. Using quantitative pathology image analysis, we extracted morphological features from H&E stained sections of formalin fixed paraffin embedded (FFPE) tumor tissues. A prediction model for patient prognosis was developed using tumor tissue pathology images from a cohort of 91 stage I lung ADC patients from the Chinese Academy of Medical Sciences (CAMS), and validated in ADC patients from the National Lung Screening Trial (NLST), and the UT Special Program of Research Excellence (SPORE) cohort. The morphological features that are associated with patient survival in the training dataset from the CAMS cohort were used to develop a prognostic model, which was independently validated in both the NLST (n = 185) and the SPORE (n = 111) cohorts. The association between predicted risk and overall survival was significant for both the NLST (Hazard Ratio (HR) = 2.20, pv = 0.01) and the SPORE cohorts (HR = 2.15 and pv = 0.044), respectively, after adjusting for key clinical variables. Furthermore, the model also predicted the prognosis of patients with stage I ADC in both the NLST (n = 123, pv = 0.0089) and SPORE (n = 68, pv = 0.032) cohorts. The results indicate that the pathology image-based model predicts the prognosis of ADC patients across independent cohorts.

## Introduction

Early diagnosis and accurate staging are among the key challenges for lung cancer patient care^1^. Patients’ survival outcomes vary substantially even within the same histology subtype and pathological stage, which is partially attributed to the highly heterogeneous nature of tumor cells and their close interaction with the diverse tumor microenvironment^2,3^. Recently, different technologies and methods have been developed to stratify cancer patients based on their molecular profiles^4,5^ or histopathological factors^6,7^, in order to facilitate personalized treatment of individual patients. Formalin fixed paraffin embedded (FFPE) tumor tissue slides provide a vast amount of information about the tumor and its surrounding microenvironment^8^; however, their potential for cancer diagnosis and treatment planning is still far from being fully explored. Currently, H&E stained tumor tissue slide scanning is becoming a routine clinical procedure. Recently, we^9^ and Yu *et al*.^10^ have demonstrated that pathology image analysis could be a promising tool to assist pathologists in lung cancer diagnosis and prognosis. However, both studies trained and validated the model using The Cancer Genome Atlas (TCGA) cohort alone. Since pathology images and patients from different cohorts may display different characteristics, in order to test the generalizability of the model, it is essential to evaluate the performance of a predictive model across multiple independent cohorts. In this study, we developed a pathology image-based prognostic model for lung adenocarcinoma (ADC) patients and validated the model in two independent lung ADC patient cohorts. This study established a generalized model that could be applied across different lung ADC patient cohorts.

## Materials and Methods

### Ethics approval and consent to participate

The University of Texas Southwestern Institutional Review Board granted approval for this research (IRB#: STU 072016-028). Data were collected under informed consent for study participation. Informed consent has been obtained for all study participation. All methods were performed in accordance with the relevant guidelines and regulations.

### Datasets

We acquired H&E-stained histological images and the corresponding clinical information for 91 stage I ADC patients from the Chinese Academy of Medical Sciences, China (CAMS), 185 ADC patients from the National Lung Screening Trial (NLST), and 111 ADC patients from the University of Texas Special Program of Research Excellence (SPORE) in Lung Cancer project. There are 91, 433, and 130 tissue slides for the CAMS, NLST and SPORE cohorts, respectively. When a patient had multiple tissue slides, the summarized value of the morphological features from multiple slides was averaged to represent the value in the patient for further statistical analyses. All tumor tissue slides are FFPE and were scanned at ×20 or ×40 magnifications. Our pathologists, Drs. Lin Yang and Junya Fujimoto, manually inspected the tissue slide images, and images with low image quality were removed from further analysis. The images captured at X40 were normalized to X20 using the method described in our previous study^9^. The characteristics of patients from different cohorts are summarized in Table 1.

**Table 1:** Patient Data Summary.

Cohort		CAMS	NLST	SPORE
Number of Patients		91	185	111
Number of Slides (Tumor)		95	357	129
Age at Diagnosis (Years) Median [LQ-HQ]		60 [55–67]	64 [60–68]	64 [58–72]
Follow-up (Years) Median [LQ-HQ]		5.0 [4.0–6.0]	6.6 [5.3–6.9]	3.6 [2.0–5.2]
Vital Status (%)	Alive	67 (73.6)	122 (65.9)	75 (67.6)
	Deceased	24 (26.4)	63 (34.1)	36 (32.4)
Gender (%)	Male	42 (46.2)	103 (55.7)	56 (50.5)
	Female	49 (53.8)	82 (44.3)	55 (49.5)
Cancer Stage (%)	I	91 (100.0)	123 (66.5)	68 (61.3)
	II	0 (0.0)	19 (10.3)	17 (15.3)
	III	0 (0.0)	31 (16.8)	24 (21.6)
	IV	0 (0.0)	12 (6.5)	1 (0.9)
	NA	0 (0.0)	0 (0.0)	1 (0.9)
Smoking Status (%)	Smoker	37 (40.7)	103 (55.7)	97 (87.4)
	Non-Smoker	54 (59.3)	82 (44.3)	13 (11.7)
	NA	0 (0.0)	0 (0.0)	1 (0.9)

Summary of the number of histological slides and patient clinical information in our study. LQ, the lower quartile, 25th percentile; HQ, the higher quartile, 75th percentile; NA, not available.

### Extract Morphological Features

Using the method described in our previous study^9^, morphological features for each image slide were extracted using CellProfiler^11,12^ software by choosing different analyses modules. These features include global features such as tissue texture and granularity, as well as cell nuclear-based features such as the size, shape, distribution, texture and neighboring architecture of nuclei. These features covered comprehensive morphological information provided by the histological images. The average signal was taken for patients with multiple image slides.

### Prognostic Model Development and Validation

Since all the pathology images and clinical information from the CAMS cohort had been strictly reviewed and assessed by a pathologist, Dr. Lin Yang, this cohort was used as the training set to develop a pathology image-based prognostic model for lung ADC patients. The morphological features were first screened by their association with patients’ survival using a univariate Cox proportional hazards regression model. Morphological features that were significantly associated (*Z score* <−*2 or* >*2*) with patients’ overall survival were selected to build a prognostic model using the random survival forest method^12^. The model was then validated in ADC patients from the NLST and SPORE cohorts, respectively. Using the risk scores assigned by the model, the patients were separated into high- and low-risk groups by the median risk score in each of the two testing sets.

### Statistical Analysis

The survival curve for each group was estimated by Kaplan-Meier method. The differences in the overall survival outcomes between high- and low-risk groups were compared using the log-rank test. Multivariate Cox proportional hazards models were used to determine the association between predicted risk groups and overall survival after adjusting for key clinical variables, including age, sex, smoking status, grade, race and stage. All the analyses were performed with R^10^ version 3.4.1.

## Results

### Extracted Morphological Features are Associated with Patients’ Survival Outcome

In total, 943 morphological features were extracted from H&E stained tumor tissue images. Among these morphological features, the top 15 features were significantly associated with patients’ survival outcome in the CAMS cohort (Table 2). Top features with the most significant Z scores were enriched in the categories of “Tissue Granularity”, “Nuclei Texture” and “Nuclei Size Shape”. Some of the features showed elevated levels of measurement in the high-risk group, whereas others showed the opposite pattern.

**Table 2:** Selected Morphological Features for Predictive Model.

Feature	Category	Zscore
Granularity_14_MaskedHema	Tissue_Granularity	−2.01
Granularity_7_MaskedEosin	Tissue_Granularity	−2.06
Mean_Nuclei_AreaShape_Zernike_2_2	Nuclei_Size_Shape	−2.65
Mean_Nuclei_AreaShape_Zernike_4_4	Nuclei_Size_Shape	−2.44
Mean_Nuclei_Texture_Contrast_Inverted_3_0	Nuclei_Texture	−2.27
Mean_Nuclei_Texture_Contrast_Inverted_3_135	Nuclei_Texture	−2.28
Mean_Nuclei_Texture_Contrast_Inverted_3_45	Nuclei_Texture	−2.44
Mean_Nuclei_Texture_Correlation_Inverted_3_45	Nuclei_Texture	2.52
Mean_Nuclei_Texture_InverseDifferenceMoment_Inverted_3_135	Nuclei_Texture	2.01
Mean_Nuclei_Texture_InverseDifferenceMoment_Inverted_3_45	Nuclei_Texture	2.25
Mean_Nuclei_Texture_Variance_Inverted_3_90	Nuclei_Texture	−2.07
Mean_Tissue_Texture_InfoMeas2_Inverted_80_135	Tissue_Texture	−2.6
Mean_Tissue_Texture_InfoMeas2_MaskedEosin_80_135	Tissue_Texture	−2.03
Mean_Tissue_Texture_InfoMeas2_MaskedEosin_80_45	Tissue_Texture	−2.11
Texture_InfoMeas2_Inverted_20_90	Tissue_Texture	−2.03

The 15 morphological features which were used in the predicative model in classifying low- and high-risk ADC patients. Z scores by univariate Cox proportional hazard analysis in CAMS ADC patients and morphological categories were reported for each feature.

### Predictive Model is Robust in Different Cohorts

**Figure 1:**
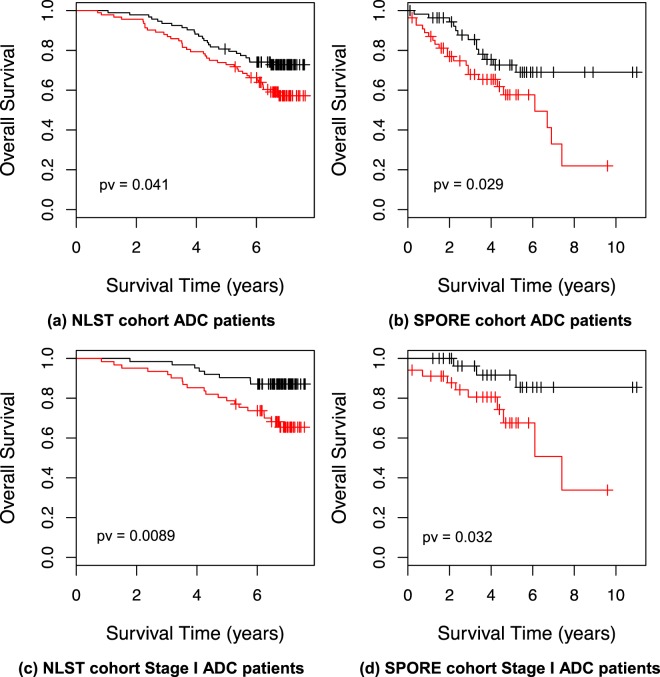
Kaplan-Meier survival curves for predicted high- and low-risk ADC patients. Using the risk score assigned by the model, the ADC patients were separated into high- and low-risk groups in (**a**) NLST cohort ADC patients, (**b**) SPORE cohort ADC patients, (**c**) NLST cohort Stage I ADC patients, (**d**) SPORE cohort Stage I ADC patients,. Kaplan-Meier survival curves were created for each risk group. The performance of the predictive model was evaluated by a log-rank test. Black line: predicted low-risk group. Red line: predicted high-risk group.

A prognostic model was developed from the CAMS patient cohort using the 15 top features as predictors. The model was then validated in both the NLST and SPORE patient cohorts. The model separated the patients in each test cohort into high- and low-risk groups. The patients in the predicted high-risk group showed a significantly worse survival than those in the predicted low-risk group, in the NLST dataset (pv = 0.0406) and SPORE dataset (pv = 0.0288), respectively. In the NLST dataset, the 5 year survival rate for the group with low risk scores was 81%, with 95% confident interval (CI) = [73–89%] versus 73% (95% CI = [64–83%]) for the group with high risk scores. In the SPORE dataset, the 5 year survival rate for the group with low risk scores was 73% (95% CI = [60–87%]) versus 58% (95% CI = [44–76%]) for the group with high risk scores (Fig. 1a,b). In multivariate analysis adjusting for clinical variables, including age, sex, smoking status, grade, race and stage (Tables 3 and 4), the association between predicted risk group and overall survival was significant for both the NLST cohort, with HR = 2.20 (predicted high-risk vs. low-risk group) and pv = 0.01, and the SPORE cohort with HR = 2.15 and pv = 0.044. Furthermore, the model predicted the prognosis of patients with stage I ADC in both the NLST (n = 123, pv = 0.0089) and the SPORE (n = 68, pv = 0.032) cohorts (Fig. 1c,d).

**Table 3:** Multivariate analysis for NLST cohort.

	HR	pv
Predicted risk group	2.20	0.010
Age	1.01	0.65
Gender (Male vs. Female)	0.72	0.27
Smoke (Yes vs. No)	1.26	0.43
Stage II vs. I	1.19	0.69
Stage III vs. I	4.04	<0.001
Stage IV vs. I	2.97	0.095
Grade 2 vs. 1	2.15	0.17
Grade 3 vs. 1	2.91	0.053
Grade 4 vs. 1	<0.01	1.00

**Table 4:** Multivariate analysis for SPORE cohort.

	HR	pv
Predicted risk group	2.15	0.044
Age	0.99	0.63
Gender (Male vs. Female)	1.19	0.64
Smoke (Yes vs. No)	1.35	0.71
Stage II vs. I	2.86	0.019
Stage III vs. I	3.36	0.005
Stage IV vs. I	57.4	0.004
Race: African American vs. Caucasian	0.85	0.83
Race: Asian vs. Caucasian	<0.01	1.00
Race: Hispanic vs. Caucasian	3.25	0.29

## Discussion

Because of the lack of standard guidelines for pathology images, images from different cohorts may vary substantially regarding the slide thickness, sectioning, staining quality and scanning magnitude. Patients in different cohorts may also display different demographic and clinical characteristics. It is essential to test the generalizability of such prognostic models by evaluating the prediction performance across multiple independent test cohorts. In this study, we have successfully validated the H&E stained tumor pathology image-based prognostic models in two independent cohorts, demonstrating the feasibility of integrating such analysis into real medical practice to assist pathologists in cancer diagnosis. Obtaining good quality and highly representative image data from patients may further improve the predication accuracy, which urges a demand for standard guidelines for pathology image acquisition and processing in the field.

## Data Availability

The data that support the findings of this study are available from the University of Texas Special Program of Research Excellence (SPORE) in Lung Cancer, National Cancer Center/Cancer Hospital and Peking Union Medical College, China, but restrictions apply to the availability of these data. Data are available from the authors upon reasonable request. Pathology images of the NLST cohort are available online at the NLST website (https://biometry.nci.nih.gov/cdas/nlst/).
